# Pyoderma Gangrenosum, Acne, and Hidradenitis Suppurativa Syndrome: A Case Report and Literature Review

**DOI:** 10.3389/fmed.2022.856786

**Published:** 2022-03-24

**Authors:** Jundong Huang, Lemuel Shui-Lun Tsang, Wei Shi, Ji Li

**Affiliations:** ^1^Department of Dermatology, Xiangya Hospital, Central South University, Changsha, China; ^2^College of Medicine, University of Tennessee Health Science Center, Memphis, TN, United States

**Keywords:** PASH syndrome, pyoderma gangrenosum, hidradenitis suppurativa, autoinflammatory syndrome, neutrophilic dermatitis

## Abstract

Pyoderma gangrenosum, acne, and hidradenitis suppurativa syndrome is a rare inflammatory disease characterized by pyoderma gangrenosum (PG), mild to severe facial acne, and hidradenitis suppurativa (HS). It only affects the skin and represents cutaneous characteristics of a spectrum of autoinflammation. Lack of pyogenic sterile arthritis (PA) distinguishes the pyoderma gangrenosum, acne, and hidradenitis suppurativa (PASH) syndrome from pyogenic arthritis, pyoderma gangrenosum, acne, and hidradenitis suppurativa (PA-PASH), pyoderma gangrenosum, acne, hidradenitis suppurtiva, and ankylosing spondylitis (PASS), and pyogenic arthritis, pyoderma gangrenosum, and acne (PAPA) syndromes. The exact etiology and pathogenesis of PASH syndrome remain unknown. Both PG and HS are contained in the spectrum of neutrophilic dermatitis, which is considered as an autoinflammatory syndrome. From a pathophysiological point of view, they show similar mechanisms, including neutrophil-rich cutaneous infiltration and overexpression of the interleukin-1 (IL-1) family. These findings provide guidance for these intractable diseases. In this review, we described a case of PASH syndrome in a patient who initially failed to respond to immunosuppressive treatment but responded to a combination of colchicine and thalidomide. We reviewed the relevant literature that focuses on PASH syndrome management.

## Introduction

The term “autoinflammatory diseases (AIDs)” was first proposed in 1999 to describe autosomal dominant periodic fever syndromes (1). Traditionally, it represents a group of hereditary recurrent non-invasive inflammatory diseases characterized by a dysfunction or hyperactivation of the innate immune system (lack of autoreactive T-cells and autoantibody production), with mutations in single genes involved in inflammation (2). As the study of AIDs deepens, it was found that such antigen-independent overactivation of the immune system played a key role in a variety of inflammatory skin diseases (3–5). A consistent feature of those disorders is neutrophil-rich cutaneous infiltration without evidence of infection. Hence, AIDs currently represent a rising group of inflammatory conditions, such as PASH syndrome, that extend beyond monogenic diseases.

Pyoderma gangrenosum, acne, and hidradenitis suppurativa (PASH) syndrome is a rare autoinflammatory dermatosis associating pyoderma gangrenosum (PG), mild to severe facial acne, and hidradenitis suppurativa (HS). Distinct genetic mutations and differences in clinical phenotypes distinguish PASH syndrome from other AIDs (6). The exact etiology and pathogenesis of PASH syndrome remain unknown. Marzano et al. (7) analyzed several patients with PASH syndrome and found the expressions of interleukin-1 beta (IL-1β) and its receptors to be remarkably higher in local skin lesions than in controls, but serum levels of the inflammatory cytokines were within normal limits. Mutations causing defective inflammasome function have been reported with PASH syndrome that increases IL-1β to stimulate innate immunity and neutrophil recruitment (8). These findings demonstrate that PASH syndrome belongs to the spectrum of IL-1-driven AIDs. Patients with PASH syndrome are treated in a variety of ways, with individual differences in efficacy. Corticosteroids combined with other immunosuppressants are usually considered first-line therapy. In this review, we described a case of PASH syndrome in a patient who failed immunosuppressive treatment initially but responded to a combination of colchicine and thalidomide. We reviewed the relevant literature that focuses on PASH syndrome management.

## Case Presentation

**FIGURE 1:**
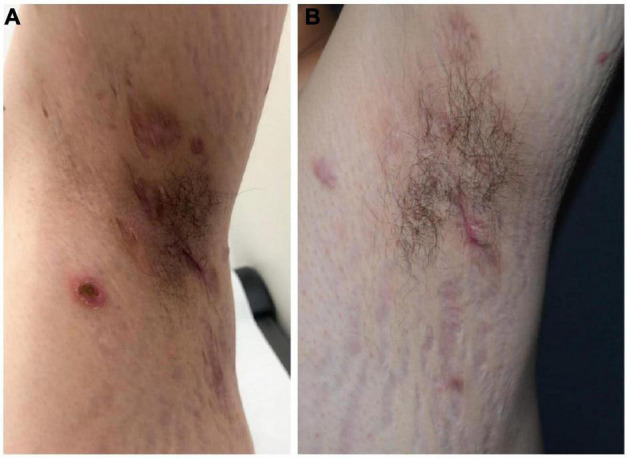
Hidradenitis suppurativa before **(A)** and following combination therapy **(B)**.

**FIGURE 2:**
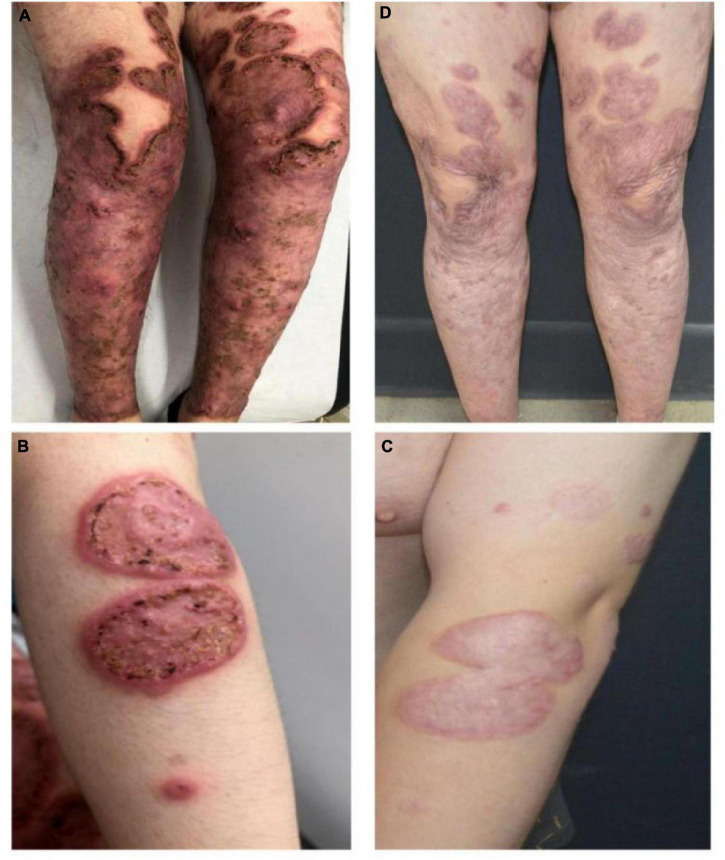
Pyoderma gangrenosum before **(A,B)** and following combination therapy **(C,D)**, the healed ulcer formed atrophic cribriform scars.

A 20-year-old man of Asian origin was referred to our hospital in July 2020 with a 9-year history of recurrent painful ulceration of both legs and aggravation during the previous few months. The patient also reported a history of recurrent draining sinuses and abscesses in the axillary and genitofemoral regions, as well as severe and scarring nodular acne of the face since puberty. Dermatological examination revealed papulopustules and sinus tracts in his axillae with purulent secretion upon palpation (Figure 1A). On his trunk and limbs, especially the lower extremities, diffuse and geographic skin ulcers surrounded by an undermined margin with apparent ridged erythema were found (Figures 2A,B). Histological examination showed pseudo-epitheliomatous hyperplasia with epidermal neutrophilic abscess formation and intradermal granuloma formation with extensive infiltration of neutrophils, which was consistent with the diagnosis of vegetative PG. PAS and acid-fast staining were negative.

Routine and immunological laboratory tests were within normal limits except for mild anemia. Considering the potential links between PG and inflammatory bowel disease or hematological diseases, serum and urine immunofixation electrophoresis and fecal calprotectin tests were conducted with negative results. A computed tomography (CT) scan of the thorax and abdominal ultrasound showed no obvious abnormalities. The patient was otherwise in good health. He denied trauma and proceeding or concurrent illnesses. His family history was unremarkable for inflammatory pathologies. Genetic testing of proline-serine-threonine phosphatase-interacting protein 1 (PSTPIP1), nicastrin (NCSTN), NOD-like receptor family pyrin domain containing 3 (NLRP3), and mediterranean fever (MEFV) genes revealed no mutations.

**FIGURE 3:**
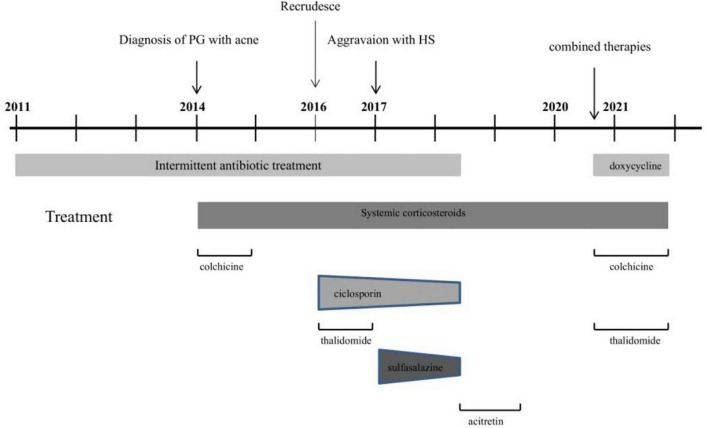
Course of disease in PASH patient from 2011 to 2021.

In view of the clinical, laboratory, and histopathological findings, we made a diagnosis of PASH syndrome. The patient had tried a variety of drugs regimens to unsatisfactory effect before coming to our clinic (Figure 3). Since the diagnosis of PG in 2014, he had been receiving oral corticosteroid therapy (initial dose prednisone 40 mg per day). The therapeutic regimen was incipiently effective, with most of his lesions subsiding after 1 month of administration. Prednisone was subsequently tapered over a year to 5 mg. However, the symptoms recurred after 2 years of maintenance; at this time, glucocorticoids alone failed to control the progression of the disease. Therefore, the patient was treated with oral corticosteroids in combination with other immunosuppressants, including sulfasalazine and cyclosporine, with only partial resolution. Attempts to add acitretin were met with failure as well. During this period, he was hospitalized several times due to secondary infection and aggravation of the illness. Until visiting our clinic, the patient was still taking oral prednisone 10 mg per day. In consideration of a therapeutic strategy targeted against all three entities (PG, HS, acne), the patient was treated with a combination of 40 mg of prednisone per day, 50 mg of thalidomide per day, 0.5 mg of colchicine two times per day, 0.2 g of doxycycline per day, and daily topical application of corticosteroids. Significant pain relief and the dramatic response of the skin lesions were observed during his second visit half a month later. Complete healing of the skin ulceration of both legs (Figures 2C,D) and remission of the facial acne and hidradenitis suppurativa (Figure 1B) were achieved within half a year. Subsequently, prednisone was tapered down to 5 mg per day within half a year, and to date, no obvious recurrence has been observed. The patient has given his consent for his case to be reported.

## Discussion

Pyoderma gangrenosum, acne, and hidradenitis suppurativa syndrome is a rare inflammatory disease characterized by PG, mild to severe facial acne, and HS. It was first described as a new entity in 2012; however, Hsiao et al. (9) had previously described this clinical phenotype. Prevalence estimates of PASH syndrome are lacking, but its associated conditions, PG and HS, have a prevalence of 0.0058% and a higher worldwide prevalence ranging from 0.3 to 1.4%, respectively (10, 11). In contrast to other autoinflammatory syndromes within the spectrum, PASH syndrome seems to only affect the skin organ and may have a wide array of genetic changes, so the diagnosis of PASH syndromes is largely based on typical clinical presentation. Both PG and HS are contained in the spectrum of neutrophilic dermatitis, and both can occur as either idiopathic diseases or syndromic manifestations. Interestingly, the coexistence of PG and HS may indicate poor response to traditional treatment options or does not achieve sustained remission. The initial symptom of our patient was painful ulceration of both legs, which rapidly responded to prednisone. However, with the recurrence of PG and the emergence of HS, glucocorticoids alone or even combined with immunosuppressants failed to provide complete relief.

As mentioned before, PASH syndrome is a heterogeneous disease. Although mutations in PSTPIP1 (an increase in the number of CCTG repeats in the PSTPIP1 promoter), PSENEN, and NCSTN have been identified in a portion of patients with PASH (12–17), the genetic background of PASH is still unclear. Genes involved in other similarly related inflammatory diseases, with which PASH syndrome shares common clinical characteristics, seem to be potential candidates. Just as there is a documented relationship between neutrophilic dermatosis and inflammatory bowel disease or malignancy, there are reports of PASH syndrome occurring in association with both of these (18, 19). It is worth noting that most patients with PASH syndrome were reported to be overweight, which is also found in PG and HS. Hence, we recommend genetic testing and thorough examination for every patient diagnosed with PASH syndrome.

With a limited number of reported cases, there are no defined treatment recommendations for PASH syndrome. Often, treatment is directed at the management of PG and HS, including wound care, topical and intralesional therapies (corticosteroids, tacrolimus, and photodynamic therapy), oral antibiotics (doxycyline, rifampin, moxifloxacin, metronidazole, amoxicillin, linezolid, etc.), traditional immunosuppressants (corticosteroids, cyclosporine, sulfasalazine, etc.), immunomodulators (thalidomide and dapsone), biologics (anti-TNF, anti-IL-1, anti-IL-17, and anti-IL-23), and surgical procedures (3, 20–27). Moreover, any proposed therapeutic strategy should address all three comprising entities as effectively as possible, and combined therapies are strongly recommended. Our general approach to treating common presentations of PASH syndrome is reviewed as follows.

Firstly, intensive lifestyle modifications focusing on weight reduction and smoking cessation can be beneficial in the treatment of PASH syndrome. For us, it is the preferred option to prescribe glucocorticoids and/or cyclosporine for timely relief of the pain and wound exacerbation. After beginning therapy, the clinician should reappraise the patient’s response to treatment within 1–3 weeks. If complete relief is not achieved, biologics or other compatible steroid-sparing agents should be considered. If there is no improvement after the above aggressive treatment, the clinician should reconsider other possible diagnoses. Furthermore, Ead et al. (28) shared two cases indicating that PASH syndrome may be a biofilm disease (a dysregulation of the host microbiota causing a persistent inflammatory condition) and emphasized the importance of antibiotic use and wound care. Frequent debridement of the wound or surgical procedures is generally considered to be avoided during the active phase of the disease with concerns for pathergic response. However, some clinical case reports (29) suggest that Negative Pressure Wound Therapy (NPWT) and Split Thickness Skin Grafts (STSG) may have surprising therapeutic effects in the early treatment of PG when combined with immunosuppressors, especially for patients with large wounds or high susceptibility to infection. While potent topical corticosteroids are often used to treat PG and HS, evidence for their efficacy in PASH is limited. We typically use them as an important adjunctive therapy and tend to taper and discontinue them over the course of 3–4 months. There is accumulating evidence indicating that biologics, particularly TNF inhibitors, have surprising efficacy when conventional immunosuppressive therapies fail to provide satisfactory results or in patients with severe organ dysfunction (30). The JAK-STAT pathway regulates signaling for multiple inflammation-relevant mediators and has been found to be associated with PG. In this vein, successful PG therapy with a JAK inhibitor was described recently (31–33). Therefore, JAK inhibitors may be a potential option consideration for PASH treatment. Colchicine has been known as an affordable, well-tolerated treatment for gout for thousands of years, which is derived from the bulb-like corms of the Colchicum autumnale plant. Studies have found that colchicine can impair neutrophil function and decrease the levels of the inflammatory cytokines (IL-1β, IFN-γ, IL-18, and IL-6) (34). Therefore, it is frequently utilized for the treatment of many inflammatory diseases, especially for those patients who cannot receive conventional immunosuppressive therapy or biologics due to contraindications such as tuberculosis or HBV infection (35). Furthermore, thalidomide and doxycycline are widely used in inflammatory skin diseases due to their potent anti-neutrophil, immunomodulatory, and anti-inflammatory cytokine activity (36, 37). In this case, based on our previous experience with the treatment of PG and HS, we implemented a combination of prednisone, thalidomide, colchicine, and doxycycline with daily topical application of corticosteroids. Complete healing of skin ulceration of both legs and remission of the facial acne and HS were achieved dramatically. Aside from the weight gain caused by oral corticosteroids, no side effects have been observed so far.

More study is needed to fully explore the pathological mechanism for this rare disease and to find more effective treatment.

## Concluding Remarks

In conclusion, PASH syndrome is a distinct entity that belongs in the spectrum of AIDs. A comprehensive and radical approach is necessary when it comes to treatment. More controlled studies with long-term follow-up are needed to confirm the efficacy of these combined therapies.

## Data Availability Statement

The original contributions presented in the study are included in the article/Supplementary Material, further inquiries can be directed to the corresponding author/s.

## Ethics statement

Written informed consent was obtained from the participant for the publication of any potentially identifiable images in this case report.

## Author Contributions

JH collected the clinical data and drafted the manuscript. LT, WS, and JL read and revised the manuscript. All authors contributed to the article and approved the submitted version and final manuscript.

## Conflict of Interest

The authors declare that the research was conducted in the absence of any commercial or financial relationships that could be construed as a potential conflict of interest.

## Publisher’s Note

All claims expressed in this article are solely those of the authors and do not necessarily represent those of their affiliated organizations, or those of the publisher, the editors and the reviewers. Any product that may be evaluated in this article, or claim that may be made by its manufacturer, is not guaranteed or endorsed by the publisher.

## References

[1] McDermott MF Aksentijevich I Galon J McDermott EM Ogunkolade BW Centola M Germline mutations in the extracellular domains of the 55 kDa TNF receptor, TNFR1, define a family of dominantly inherited autoinflammatory syndromes. *Cell.* (1999) 97:133–44. 10.1016/s0092-8674(00)80721-7 10199409

[2] Di Donato G d’Angelo DM Breda L Chiarelli F. Monogenic autoinflammatory diseases: state of the art and future perspectives. *Int J Mol Sci.* (2021) 22:6360. 10.3390/ijms22126360 34198614 PMC8232320

[3] Maverakis E Marzano AV Le ST Callen JP Brüggen MC Guenova E Pyoderma gangrenosum. *Nat Rev Dis Primers.* (2020) 6:81. 33033263 10.1038/s41572-020-0213-x

[4] Goldburg SR Strober BE Payette MJ. Hidradenitis suppurativa: epidemiology, clinical presentation, and pathogenesis. *J Am Acad Dermatol.* (2020) 82:1045–58. 31604104 10.1016/j.jaad.2019.08.090

[5] Li ZJ Choi DK Sohn KC Seo MS Lee HE Lee Y Propionibacterium acnes activates the NLRP3 inflammasome in human sebocytes. *J Invest Dermatol.* (2014) 134:2747–56. 10.1038/jid.2014.221 24820890

[6] Cugno M Borghi A Marzano AV. PAPA, PASH and PAPASH syndromes: pathophysiology, presentation and treatment. *Am J Clin Dermatol.* (2017) 18:555–62. 10.1007/s40257-017-0265-1 28236224

[7] Marzano AV Ceccherini I Gattorno M Fanoni D Caroli F Rusmini M Association of pyoderma gangrenosum, acne, and suppurative hidradenitis (PASH) shares genetic and cytokine profiles with other autoinflammatory diseases. *Medicine (Baltimore).* (2014) 93:e187. 10.1097/md.0000000000000187 25501066 PMC4602806

[8] Jfri AH O’Brien EA Litvinov IV Alavi A Netchiporouk E. Hidradenitis suppurativa: comprehensive review of predisposing genetic mutations and changes. *J Cutan Med Surg.* (2019) 23:519–27. 10.1177/1203475419852049 31167568

[9] Hsiao JL Antaya RJ Berger T Maurer T Shinkai K Leslie KS. Hidradenitis suppurativa and concomitant pyoderma gangrenosum: a case series and literature review. *Arch Dermatol.* (2010) 146:1265–70. 10.1001/archdermatol.2010.328 21079064

[10] Jfri A Nassim D O’Brien E Gulliver W Nikolakis G Zouboulis CC. Prevalence of Hidradenitis suppurativa: a systematic review and meta-regression analysis. *JAMA Dermatol.* (2021) 157:924–31. 10.1001/jamadermatol.2021.1677 34037678 PMC8156162

[11] Xu A Balgobind A Strunk A Garg A Alloo A. Prevalence estimates for pyoderma gangrenosum in the United States: an age- and sex-adjusted population analysis. *J Am Acad Dermatol.* (2020) 83:425–9. 10.1016/j.jaad.2019.08.001 31400451

[12] Sonbol H Duchatelet S Miskinyte S Bonsang B Hovnanian A Misery L. PASH syndrome (pyoderma gangrenosum, acne and hidradenitis suppurativa): a disease with genetic heterogeneity. *Br J Dermatol.* (2018) 178:e17–8. 10.1111/bjd.15740 28626985

[13] Calderón-Castrat X Bancalari-Díaz D Román-Curto C Romo-Melgar A Amorós-Cerdán D Alcaraz-Mas LA PSTPIP1 gene mutation in a pyoderma gangrenosum, acne and suppurative hidradenitis (PASH) syndrome. *Br J Dermatol.* (2016) 175:194–8. 10.1111/bjd.14383 26713508

[14] Duchatelet S Miskinyte S Join-Lambert O Ungeheuer MN Francès C Nassif A First nicastrin mutation in PASH (pyoderma gangrenosum, acne and suppurative hidradenitis) syndrome. *Br J Dermatol.* (2015) 173:610–2. 10.1111/bjd.13668 25601011

[15] Braun-Falco M Kovnerystyy O Lohse P Ruzicka T. Pyoderma gangrenosum, acne, and suppurative hidradenitis (PASH)–a new autoinflammatory syndrome distinct from PAPA syndrome. *J Am Acad Dermatol.* (2012) 66:409–15. 10.1016/j.jaad.2010.12.025 21745697

[16] Marzano AV Damiani G Ceccherini I Berti E Gattorno M Cugno M. Autoinflammation in pyoderma gangrenosum and its syndromic form (pyoderma gangrenosum, acne and suppurative hidradenitis). *Br J Dermatol.* (2017) 176:1588–98. 10.1111/bjd.15226 27943240

[17] Zhang X He Y Xu H Wang B. First PSENEN mutation in PASH syndrome. *J Dermatol.* (2020) 47:1335–7. 10.1111/1346-8138.15527 32770559

[18] Maione V Perantoni M Caravello S Zambelli C Calzavara-Pinton P. A case of PASH syndrome associated to testicular cancer. *Dermatol Ther.* (2021) 34:e14763. 10.1111/dth.14763 33405308

[19] Murphy B Morrison G Podmore P. Successful use of adalimumab to treat pyoderma gangrenosum, acne and suppurative hidradenitis (PASH syndrome) following colectomy in ulcerative colitis. *Int J Colorectal Dis.* (2015) 30:1139–40. 10.1007/s00384-014-2110-9 25564349

[20] Zagaria O Ruggiero A Fabbrocini G Gallo L Romanelli M Marasca C. Wound care, adalimumab, and multidisciplinary approach in a patient affected by PASH syndrome. *Int Wound J.* (2020) 17:1528–31. 10.1111/iwj.13403 32441488 PMC7948612

[21] Kok Y Nicolopoulos J Varigos G Howard A Dolianitis C. Tildrakizumab in the treatment of PASH syndrome: a potential novel therapeutic target. *Australas J Dermatol.* (2020) 61:e373–4. 10.1111/ajd.13285 32285437

[22] Jennings L Molloy O Quinlan C Kelly G O’Kane M. Treatment of pyoderma gangrenosum, acne, suppurative hidradenitis (PASH) with weight-based anakinra dosing in a hepatitis B carrier. *Int J Dermatol.* (2017) 56:e128–9. 10.1111/ijd.13528 28239847

[23] Staub J Pfannschmidt N Strohal R Braun-Falco M Lohse P Goerdt S Successful treatment of PASH syndrome with infliximab, cyclosporine and dapsone. *J Eur Acad Dermatol Venereol.* (2015) 29:2243–7. 10.1111/jdv.12765 25352307

[24] Hatanaka M Fujii K Kanekura T. Successful treatment of pyoderma gangrenosum, acne, and suppurative hidradenitis syndrome with granulocyte and monocyte adsorption apheresis. *J Dermatol.* (2021) 48:e376–7. 10.1111/1346-8138.15946 33991008

[25] Gul MI Singam V Hanson C Neill BC Aires DJ Rajpara AN. Remission of refractory PASH syndrome using Ixekizumab and doxycycline. *J Drugs Dermatol.* (2020) 19:1123. 10.36849/JDD.2020.1123 33196740

[26] Saint-Georges V Peternel S Kaštelan M Brajac I. Tumor necrosis factor antagonists in the treatment of pyoderma gangrenosum, acne, and suppurative hidradenitis (PASH) syndrome. *Acta Dermatovenerol Croat.* (2018) 26:173–8. 29989876

[27] Join-Lambert O Duchatelet S Delage M Miskinyte S Coignard H Lemarchand N Remission of refractory pyoderma gangrenosum, severe acne, and hidradenitis suppurativa (PASH) syndrome using targeted antibiotic therapy in 4 patients. *J Am Acad Dermatol.* (2015) 73(5 Suppl. 1):S66–9. 10.1016/j.jaad.2015.07.040 26470620

[28] Ead JK Snyder RJ Wise J Cuffy C Jafary H Fischborn K. Is PASH syndrome a biofilm disease?: a case series and review of the literature. *Wounds.* (2018) 30:216–23. 30212364

[29] Pichler M Larcher L Holzer M Exler G Thuile T Gatscher B Surgical treatment of pyoderma gangrenosum with negative pressure wound therapy and split thickness skin grafting under adequate immunosuppression is a valuable treatment option: case series of 15 patients. *J Am Acad Dermatol.* (2016) 74:760–5. 10.1016/j.jaad.2015.09.009 26979359

[30] De Wet J Jordaan HF Kannenberg SM Tod B Glanzmann B Visser WI. Pyoderma gangrenosum, acne, and suppurative hidradenitis syndrome in end-stage renal disease successfully treated with adalimumab. *Dermatol Online J.* (2017) 23:6. 29447652

[31] Kochar B Herfarth N Mamie C Navarini AA Scharl M Herfarth HH. Tofacitinib for the treatment of pyoderma gangrenosum. *Clin Gastroenterol Hepatol.* (2019) 17:991–3. 30404036 10.1016/j.cgh.2018.10.047

[32] Gregory MH Ciorba MA Deepak P Christophi GP. Successful treatment of pyoderma gangrenosum with concomitant tofacitinib and infliximab. *Inflamm Bowel Dis.* (2019) 25:e87–8. 10.1093/ibd/izz015 30753456

[33] Nasifoglu S Heinrich B Welzel J. Successful therapy for pyoderma gangrenosum with a Janus kinase 2 inhibitor. *Br J Dermatol.* (2018) 179:504–5. 10.1111/bjd.16468 29451690

[34] Dasgeb B Kornreich D McGuinn K Okon L Brownell I Sackett DL. Colchicine: an ancient drug with novel applications. *Br J Dermatol.* (2018) 178:350–6. 10.1111/bjd.15896 28832953 PMC5812812

[35] Rao S Shi W. A case of adult-onset Still’s disease accompanied with pulmonary tuberculosis successfully treated with colchicine. *Postepy Dermatol Alergol.* (2021) 38:912–5. 10.5114/ada.2021.110105 34849145 PMC8610067

[36] Shehwaro N Langlois AL Gueutin V Gauthier M Casenave M Izzedine H. [Doxycycline or how to create new with the old?]. *Therapie.* (2014) 69:129–41. 10.2515/therapie/2013069 24926631

[37] Paravar T Lee DJ. Thalidomide: mechanisms of action. *Int Rev Immunol.* (2008) 27:111–35. 18437602 10.1080/08830180801911339

